# Emerging Concepts in the Resolution of Periodontal Inflammation: A Role for Resolvin E1

**DOI:** 10.3389/fimmu.2017.01682

**Published:** 2017-12-14

**Authors:** Maria G. Balta, Bruno G. Loos, Elena A. Nicu

**Affiliations:** ^1^Department of Periodontology, Academic Centre for Dentistry Amsterdam, Amsterdam, Netherlands; ^2^Opris Dent SRL, Sibiu, Romania

**Keywords:** Resolvin E1, periodontitis, inflammation, resolution, proresolving mediators

## Abstract

Inflammatory response is a protective biological process intended to eliminate the harmful effect of the insulting influx. Resolution of inflammation constitutes an active sequence of overlapping events mediated by specialized proresolving mediators, such as lipoxins, resolvins, protectins, and maresins, which originate from the enzymatic conversion of polyunsaturated fatty acids (PUFAs). An unresolved acute inflammatory response results in chronic inflammation, which is a leading cause of several common pathological conditions. Periodontitis is a biofilm-induced chronic inflammatory disease, which results in loss of periodontal connective tissue and alveolar bone support around the teeth, leading to tooth exfoliation. An inadequate proresolving host response may constitute a mechanism explaining the pathogenesis of periodontal disease. An emerging body of clinical and experimental evidence has focused on the underlying molecular mechanisms of resolvins and particularly Resolvin E1 (RvE1) in periodontitis. Recently, RvE1 has been directly correlated with the resolution of inflammation in periodontal disease. Herein, we provide a comprehensive overview of the literature regarding the role and possible mechanisms of action of RvE1 on different cell populations recruited in periodontal inflammation as well as its potential therapeutic implications. Along with recent data on the benefits of PUFAs supplementation in periodontal clinical parameters, we touch upon suggested future directions for research.

## Introduction

Inflammation constitutes an essential biological process of the immune host response, which is activated when the natural tissue homeostasis is disturbed after infection or injury. By design, the inflammatory process is programmed to cease, allowing the return of the affected tissue to its preinflammatory state and function. In the past, it was believed that inflammatory resolution was a passive event, which results from the dilution of chemokine gradients over time, thus reducing the chemotaxis of leukocytes to the site of injury. However, evidence from studies performed the last few decades has shown that it is a carefully orchestrated active process (1). The identification of specific proresolving pathways and lipid mediators by Serhan et al. introduced a paradigm shift and opened a new window to understanding the resolution of inflammation (2, 3). In contrast to previous considerations, it is now widely appreciated that it represents a sequence of overlapping events during which proinflammatory mediators signal the generation of specialized proresolving mediators (SPMs) or induce their receptor targets. In other words, the signals that regulate the resolution of acute inflammatory responses are tightly interrelated with the mediators, which initiate these responses (4). Thus, as it will be more thoroughly described later, the peak of the acute inflammatory response is considered the beginning of resolution, suggesting a concept where the “beginning programs the end” (5, 6).

Acting either as agonists or partial agonists at specific G-protein-coupled receptors (ChemR23, BLT1, ALX/FPR2, GPR32, and GPR18), SPMs can elicit a spectrum of cell type-specific responses that collectively block the inflammatory process (7). Critical prerequisites for inflammation to switch off are the removal of the inciting stimulus, inhibition of leukocyte trafficking, catabolism of proinflammatory mediators, phagocytosis of apoptotic cells and clearance of the lesion (8). However, for reasons that are not yet fully elucidated, one or more of the abovementioned proresolving pathways might be insufficiently engaged, allowing for the acute inflammatory response to continue undisturbed and to result in chronic pathology.

Periodontitis is a biofilm-induced chronic inflammatory disease, which results in loss of periodontal connective tissue and alveolar bone support around the teeth. Long-term studies have shown that untreated periodontal disease may result in tooth loss (9, 10).

Periodontal homeostasis can be disrupted by a variety of host- or microbe-related factors. Historically, it was believed that periodontal inflammation was mainly driven by bacterial influx. The experimental gingivitis model and extensive corroborating studies provided the first concrete evidence supporting that plaque accumulation was essential for the initiation and establishment of periodontal inflammation (11, 12). In 1994, Marsh proposed the “Ecological Plaque Hypothesis,” suggesting that an imbalance in the total microflora, due to ecological stress, allows for the overgrowth of disease-related microbes (13). This hypothesis underlined the interaction between the local environment and the composition of dental plaque. Along these lines, it has been recently demonstrated that the establishment of inflammation can induce changes in the composition of subgingival microbiota. In fact, the ensuing chronic and hyper reactive immune response provides a rich and favorable ecological system where periodontitis-associated microbiota can thrive (14, 15). Utilizing a panel of virulence factors that affect the host response mechanisms, such microbial pathogens can influence the stability and dynamics of the biofilm and induce a shift to a more pathogenic flora, thus converting a symbiotic environment to a dysbiotic one (16). Recent evidence provided by animal studies has confirmed that regulating inflammation through resolution pathways can result in a beneficial shift in the microflora and reduce the relative abundance of disease-related genera (17).

Periodontal inflammation is associated with microbial dysbiosis, but it is the host inflammatory response to the microbial challenge that is responsible for the degradation of the periodontium (14). In fact, although intended for the host defense against invading microbes, leukocytes, and polymorphonuclear neutrophils (PMNs) in particular can amplify tissue destruction *via* the release of proinflammatory mediators, reactive oxygen species (ROS) and enzymes (18). The pathogenesis of the disease is therefore characterized by an altered or aberrant host response to the present microflora, leading to immune cell-mediated self-destruction of periodontal tissues (15, 16).

Susceptibility to periodontitis, as well as a persistent inflammatory reaction, might originate from the simultaneous occurrence of various host- or microbe-related factors. Thus, an inadequate proinflammatory or proresolving host response, or an imbalance between the two, might be a mechanism contributing to the pathogenesis of the disease. Hence, controlling the fate of the proresolving pathways could have an impact on the conversion of a gingivitis case to periodontitis (19).

Periodontitis is a leukocyte-driven inflammatory disease characterized by uncontrolled inflammation (20). During the last decades, a rapidly emerging body of evidence has focused on the role of SPMs in periodontitis and has provided a window to explore the pathophysiology of this chronic inflammatory disease.

In this review, we present recent insights into the resolution of periodontal inflammation. Since most of the studies on the topic are based on work with Resolvin E1 (RvE1), we highlight *in vitro* and *in vivo* experimental findings focusing on RvE1 research. We also touch upon studies involving other SPMs when relevant to periodontal inflammation.

## Resolution of Inflammation; from Lipoxins to Resolvins

The first evidence that resolution is actively “turned on” by families of lipid mediators came with the identification of lipoxins almost four decades ago. Lipoxins are metabolites of arachidonic acid and were initially isolated from activated human leukocytes by Serhan et al. (2). Their biosynthetic pathways involve cell–cell interactions and require the action of specific lipoxygenases (LOs) (5-LO, 12-LO, and 15-LO) (21). The epimeric forms of lipoxins are additionally generated under the influence of aspirin and are also known as aspirin-triggered lipoxins (ATLs) (22). Lipoxins and ATLs serve as endogenous mediators, which play important roles in downregulating the recruitment of PMNs and uptake of apoptotic PMNs by macrophages to facilitate clearance of the inflammatory lesion (23).

Almost two decades later, the SPM family was completed by the characterization of resolvins, the aspirin-triggered forms of resolvins, protectins, and later, maresins. The mouse dorsal air pouch serves as an *in vivo* model for studying the spontaneous resolution of inflammation. Mice received injections of tumor necrosis factor alpha (TNF-α), eicosapentaenoic acid (EPA), and/or docosahexaenoic acid (DHA) combined with aspirin treatment. Exudates were collected 6 h later and were subjected to liquid chromatography tandem mass spectrometry. The analysis revealed the presence of novel hydroxy acids produced from both EPA and DHA within the inflammatory exudate. The same compounds were also generated *in vitro* from coincubation of human endothelial cells with PMNs (3, 24). The enzymatic conversion of EPA leads to the generation of E-series resolvins, whereas DHA constitutes the parent substrate for the formation of D-series resolvins, protectins, and maresins (25).

## Generation of Resolvins

The ω-3 fatty acids constitute a family of essential fats that humans are unable to synthesize *de novo*. EPA and DHA are the principal long-chain ω-3 fatty acids in the diet. Algae are the main producers of these essential fats in the ecosystem, and therefore fish, which are major algae consumers, are rich in EPA and DHA. Since the enzymatic conversion of the parent a-linolenic acid to EPA and DHA appears to be insufficient in humans, intake of these nutrients through the diet is necessary (26).

Among their biological functions, polyunsaturated fatty acids (PUFAs) have been implicated in modulating anti-inflammatory responses *via* binding to G-protein-coupled receptors on leukocytes. For instance, activation of GPR120, which is expressed on macrophages by EPA and DHA, was shown to inhibit both Toll-like receptor and TNF-α inflammatory signaling pathways (27). Apart from being structural components of biological membranes, ω-3 fatty acids are the precursors of proresolving lipid mediators. During the early phases of the acute inflammatory response, rapid changes in local blood vessel perfusion and permeability occur. These events permit the extravasation of circulating leukocytes and plasma proteins but also provide a means to deliver EPA and DHA from the circulation to the inflamed site. Indeed, SPM precursors (i.e., EPA and DHA) appear at the site of the inflammatory lesion in tandem with the extravasation of serum proteins, paralleling edema (28). Such a finding highlights the important role of edema with exudation of serum proteins as a means to effectively deliver ω-3 PUFAs to the inflamed tissue (29).

**Figure 1:**
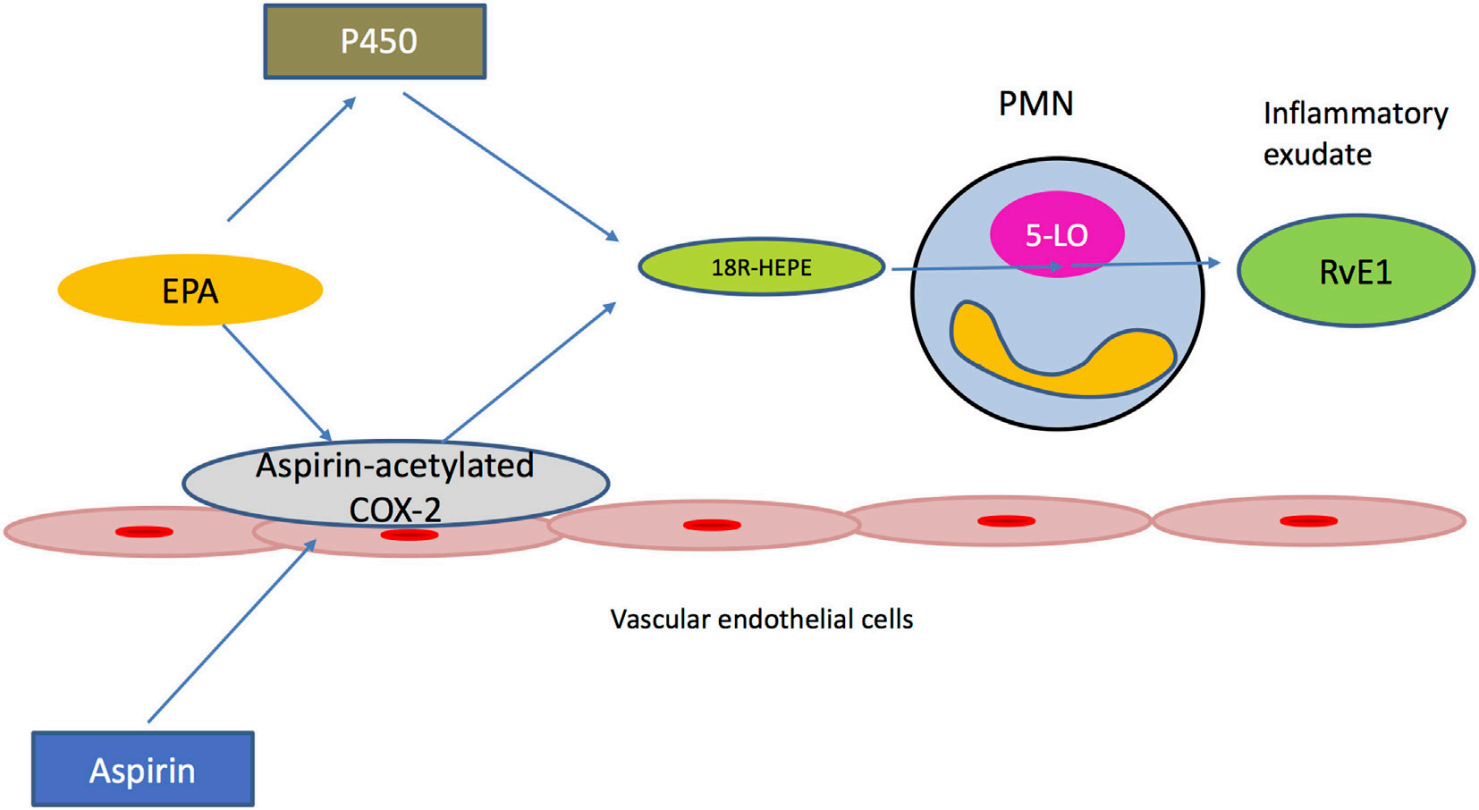
Transcellular biosynthesis of Resolvin E1 (RvE1). Edema formation delivers eicosapentaenoic acid (EPA) to the inflamed tissue. Aspirin acetylates cyclooxygenase 2 (COX2), which is present in vascular endothelial cells. The ASA-acetylated form of COX2 is still active and converts EPA to 18R-HEPE, which is then released from the endothelium. Activated polymorphonuclear neutrophils rapidly convert 18R-HEPE to Resolvin E1 (RvE1) *via* the action of 5-lipoxygenase (5-LO). Cytochrome P450, present in different microbes, can alternatively mediate the generation of 18R-HEPE, which will be converted to RvE1 by the host’s leukocytes.

One well-described pathway resulting in the generation of RvE1 involves the triggering role of aspirin (Figure 1). In fact, aspirin treatment at local sites of inflammation can acetylate cyclooxygenase 2 (COX2), which is present in vascular endothelial cells. The acetylated form of COX2 is able to convert EPA present in the inflammatory exudate to its intermediate precursor 18R-hydro (peroxy)-EPE. This is quickly reduced to 18R-HEPE, which is then released from endothelium and rapidly converted to RvE1 *via* the action of 5-LO. 5-LO is an enzyme present in activated PMNs, which are abundant in the inflammatory lesion. In this way, cell–cell interactions between activated PMNs and vascular endothelial cells within the exudate can initiate the transcellular biosynthesis of lipid mediators (30).

Resolvin E1 can also be generated *via* the microbial P450 pathway. In this case, cytochrome P450 (CYP) mono-oxygenase, which is present in different types of microbes, such as bacteria, fungi and viruses, can mediate the generation of the precursor molecule 18R-HEPE, which will be converted to RvE1 by LO-5 of the host’s leukocytes. Additionally, the same enzyme (CYP) is able to produce RvE1 by processing leukotriene B5 (LTB5), which is generated *via* the host’s 5-LO (31). Of interest, *Candida albicans* can biosynthesize RvE1 that is chemically identical to that produced by human cells. In contrast to the transcellular biosynthesis of human resolvin, RvE1 generation in *C. albicans* occurs in the absence of other cellular partners. However, it is unclear how RvE1 production is beneficial to *C. albicans*, since human PMNs show enhanced phagocytosis and hydroxyl-radical-mediated killing of *C. albicans* in the presence of RvE1 (32).

## RvE1 Receptors

Once formed, RvE1 acts on target cells in its immediate milieu in an autocrine and paracrine fashion and then is locally inactivated by site-specific metabolism (18). The action of RvE1 on these target cells is mediated *via* specific G-protein-coupled transmembrane receptors. Currently, two of these receptors, which interact with RvE1, have been identified: ChemR23 and BLT1.

Resolvin E1 acts as an agonist on ChemR23, which is mainly expressed on monocytes, macrophages, dendritic cells, and osteoblasts (OBs). Even though the data are contradictory, expression of ChemR23 at low levels on PMNs isolated from healthy individuals has been recently described. Interestingly, diabetes has been associated with an overexpression of ChemR23 on PMNs. In fact, although PMNs from healthy subjects have mainly exhibited BLT1 expression and minimal levels of functional ChemR23, PMNs from diabetics have shown an inverse coexpression of the two receptors, suggesting a dysregulated neutrophil receptor profile (33, 34). ChemR23 was named after its natural ligand, chemerin, which is reported to act as a chemoattractant and as an adipokine (35). *Via* binding to ChemR23, RvE1 regulates the migration and cytokine production of macrophages and dendritic cells (36). In addition, RvE1 appears to induce phosphorylation of two proteins (Akt and ribosomal protein S6) involved in translation (37). When ribosomal protein S6 (rS6) is phosphorylated, cells expressing ChemR23, such as macrophages, undergo growth (38). Furthermore, upon incubation with RvE1, human macrophages display enhanced phagocytosis. These findings support the hypothesis that binding of RvE1 to ChemR23 enhances phagocytosis of apoptotic PMNs by macrophages and thus promotes clearance of the inflammatory lesion, a critical step in the resolution of inflammation (37).

In addition to its action through ChemR23, RvE1 exerts its proresolving action *via* binding to another receptor, BLT1. BLT1, which is highly expressed on PMNs and osteoclasts (OCs), is a high-affinity receptor for LTB4 (39). LTB4 acts as a proinflammatory mediator; it is mainly produced by stimulated leukocytes and enhances the activation and chemotaxis of PMNs, eosinophils and macrophages (40). RvE1 has been shown to compete with LTB4 for binding to BLT1, by acting as a partial agonist while attenuating LTB4-induced proinflammatory signals. In this way, RvE1 limits LTB4-mediated leukocyte infiltration and activation, which is a critical counterregulatory step in inflammation (39). Furthermore, in the absence of LTB4, incubation with RvE1 also regulates PMN apoptosis by dampening the intracellular survival signals from other mediators, such as the PMN granule enzyme myeloperoxidase, the acute-phase protein serum amyloid A or the bacterial constituent CpG DNA (33).

## Studies on the Role of RvE1 in Periodontal Inflammation

### Effects on Leukocyte Recruitment and Extravasation

**Figure 2:**
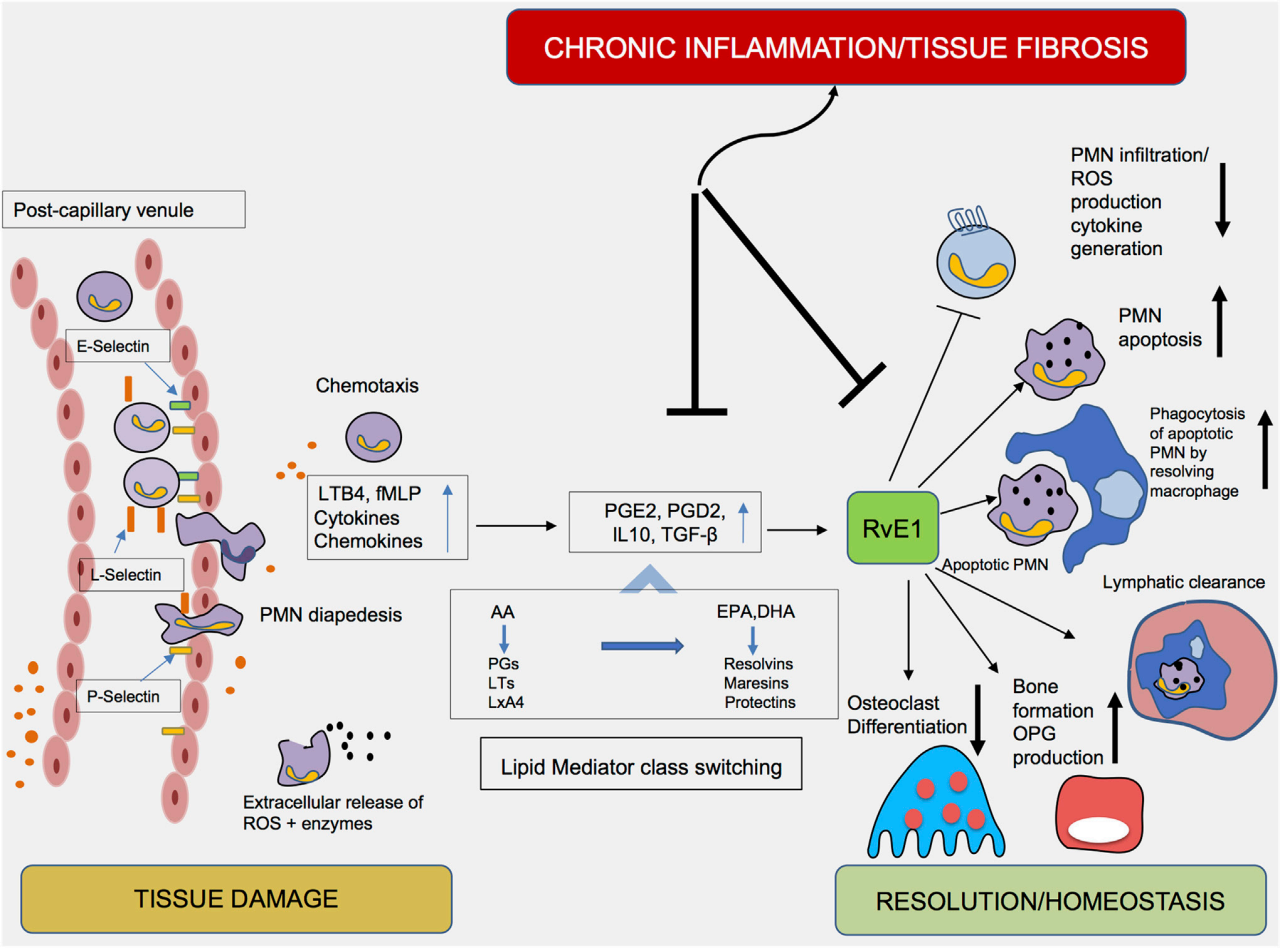
Cellular components and mediators of inflammation resolution. An increase in vascular permeability, in combination with the expression of cellular adhesion molecules (i.e., selectins and integrins), enables diapedesis of polymorphonuclear neutrophils (PMNs) to the inflamed periodontal tissue. Under the influence of chemotactic factors, PMNs transmigrate into tissues. The early stages of the inflammatory reaction are characterized by the synthesis of eicosanoids (leukotrienes and prostaglandins), which initiate and amplify PMN chemotaxis. Exposure of PMNs to agents such as PGE2 and PGD2 in the exudate initiate a shift (“lipid mediator class switching”) from proinflammatory eicosanoids toward proresolving lipid mediators, such as Resolvin E1 (RvE1). Acting either as an agonist or antagonist at certain receptors, RvE1 inhibits PMN infiltration and reactive oxygen species production, enhances PMN apoptosis and clearance by “resolving” macrophages and promotes the reduction of PMN influx, a critical prerequisite for the resolution of inflammation.

During periodontal inflammation, bacteria residing in the biofilm and certain bacterial components are recognized *via* pattern recognition receptors (PRRs). Toll-like receptors, present on host cells, detect molecules broadly shared by microorganisms; these molecules, such as lipopolysaccharides, lipoproteins, and lipoteichoic acids, are described using the term “pathogen-associated molecular patterns” (PAMPs) (41). Recognition of PAMPs, *via* binding to these PRRs, has been shown to induce the secretion of chemoattractant proteins, namely, chemokines, which in turn attract PMNs at the site of infection. Extravasated PMNs enter the gingival tissues and subsequently enter the crevice through the junctional epithelium. Their extravasation is mediated by the combined action of high- and low-affinity adhesive interactions and implicates crucial morphological changes in both PMNs and endothelial cells (42). Tissue-derived cytokines enhance endothelial adhesion molecule expression, whereas tissue-derived chemokines induce changes on leukocyte integrins, enabling them to acquire a high-affinity state (43). The initial contact, namely, “tethering” with the vascular wall and adhering to endothelial cells, is mediated by selectins and their ligands. L-selectin is expressed by leukocytes, whereas P- and E-selectins are expressed on activated endothelial cells and platelets (Figure 2). Tethering slows the speed of circulating PMNs and favors subsequent interactions mediated by integrins, which permit the firm adherence of leukocytes on endothelial cells. *Via* interactions with their endothelial counter-receptors, β2-integrins, as well as lymphocyte function-associated antigen 1, play essential roles in regulating the firm adhesion of PMNs to the endothelium (44). The abovementioned events halt the traveling PMNs on the vascular wall and enable their diapedesis into the infected tissue (45, 46).

In this way, PMNs transmigrate in large numbers into tissues and neutralize pathogens by engulfing them *via* phagocytosis, release of bactericidal proteins stored in their intracellular granules and production of ROS (47). To avoid PMN-induced tissue damage, it has been proposed that the presence of PMNs in the tissues is regulated *via* a “feedback loop.” Recruited PMNs undergo apoptosis and are consequently removed by macrophages. It has been described that phagocytosis of microbial or damaged tissue cells by PMNs stimulates their apoptosis, an essential step in resolution of inflammation. Incubation with RvE1 for 24 h has been demonstrated to facilitate phagocytosis-induced PMN apoptosis (33).

Extravasated apoptotic PMNs are cleared *via* phagocytosis by tissue macrophages. This action hampers the expression of interleukin (IL)-23, which consequently suppresses the generation of IL-17 and granulocyte colony stimulating factor and, thus, downregulates granulopoiesis in the bone marrow (44). Patients with periodontitis exhibit a higher number of PMNs and longer lived PMNs in gingival tissues, as well as reduced PMN chemotactic accuracy, compared with healthy individuals (48, 49). Fredman et al. (50) also reported elevated expression of integrins on the circulating PMNs and monocytes of localized aggressive periodontitis (LAgP) patients, indicating that the resting cells within the whole blood of these patients circulate in a primed state.

It has been suggested that the persistent recruitment of PMNs in inflamed periodontal tissues might be partially due to the inability to control the subgingival microbial challenge. There is evidence that RvE1 can, to a certain extent, counter-regulate this stage in the inflammatory reaction. Administration of RvE1 reduced the infiltration of leukocytes, primarily PMNs, by 50% in peritoneal exudates of mice with zymosan-induced peritonitis (51). This can be possibly explained by the effect of RvE1 on the expression of adhesion molecules on leukocytes. In fact, RvE1 reduces the L-selectin and integrin CD18 expression in peripheral blood PMNs and monocytes, thus impairing binding to their endothelial counterreceptors. In the same direction, administration of RvE1 resulted in 40% downregulation in leukocyte rolling, as confirmed *via* intravital microscopy (52). Additionally, *in vivo* experiments in rats with ligature-induced periodontitis showed that local treatment with RvE1 solution significantly suppressed inflammatory cell infiltration and downregulated expression of inflammation-associated genes in the gingival tissues (17).

### Effects on Platelets

Apart from their well-described functions in thrombosis and hemostasis, platelets have also been implicated in inflammation. Increasing evidence supports that platelets, including their interactions with inflammatory cells, play a crucial role in atheromatic plaque initiation, instability and rupture (53). The thrombogenic capacity of platelets depends on their ability to aggregate, a process mediated by selectins and integrins (54). Platelets from periodontitis patients demonstrate increased activation in comparison to platelets from healthy controls. In this activation process, P-selectin mediates coaggregation and inflammation by regulating cell–cell interactions between platelets, leukocytes and endothelial cells. In fact, periodontitis patients demonstrated increased plasma levels of sP-Selectin, as well as elevated binding of platelet glycoprotein IIb–IIIa complex, a recognized measure of platelet aggregation (55). Moreover, platelets from periodontitis patients exhibited increased sensitivity to activation by oral bacteria and formed increased numbers of platelet–monocyte complexes compared to healthy controls (56). Incubation of platelet-rich plasma with RvE1 blocked adenosine diphosphate (ADP)-induced platelet aggregation in healthy donors, demonstrating that RvE1 acts selectively and inhibits platelet aggregation (52). The molecular pathway behind this inhibitory action was further elucidated by Fredman et al. (54), who demonstrated that, *via* stereoselective binding to ChemR23 on platelets, RvE1 reduces P-selectin surface mobilization and induces platelet–actin polymerization. Platelet responses to ADP require the activation of two G-protein-coupled receptors, namely, P2Y1 and P2Y12 (57). In general, the intracellular pathway involves the inhibition of adenyl cyclase (*via* activation of P2Y12) and increase in intracellular Ca^2+^ (*via* activation of P2Y1). Of note, RvE1 did not alter Ca^2+^ mobilization, indicating that RvE1 exerts its counterregulatory effect by P2Y12-ADP signaling *via* ChemR23 (54).

### Effects on ROS Generation

Peripheral blood PMNs from chronic periodontitis patients exhibit excess ROS production following stimulation with bacterial stimuli (stimulated hyperreactivity), as well as in the absence of exogenous stimulation (intrinsic hyperactivity), when compared to age-/sex- and smoking-matched healthy controls (58, 59). As a result, their prolonged presence in the tissues, in combination with the release of the abovementioned molecules and tissue-degrading enzymes, can result in collateral tissue damage (60). After incubation with RvE1, superoxide generation was blocked >90% in PMNs from patients with LAgP. When incubated with increasing concentrations of RvE1 and subsequently challenged with N-formyl-methionyl-leucyl-phenylalanine, PMNs from LAgP patients and controls responded with an 80% reduction in superoxide generation (16), providing an indication that this lipid mediator counteracts the collateral tissue damage induced by PMNs in periodontal inflammation (Figure 2).

### Effects on PMNs Clearance and Generation of Cytokines

At a later stage in the inflammatory reaction, leukocytes of the monocyte/macrophage lineage are recruited to the inflammatory milieu. PMNs that have already been recruited appear to mediate this switch by releasing soluble factors that stimulate monocyte recruitment (61). Monocyte-derived macrophages replace the PMNs as the predominant leukocytes and perform an impressive repertoire of functions involving elimination of bacterial stimuli, recruitment of other cells to the site of infection, clearance of the excess PMNs, generation of cytokines/chemokines and activation of the lymphocyte-mediated adaptive immune response. In the presence of RvE1, the production of TNF-α and IL-8 by monocytes in a whole blood culture model was significantly reduced, whereas RvE1 did not trigger the generation of other cytokines, such as IL-10, interferon-γ, and IL-1β (52). Consistent with their functional role, macrophages acquire different phenotypes. In fact, they can modify their metabolic setup and polarize from a healing/growth-promoting setting (M2 phenotype) to a killing/inhibitory function (M1 phenotype) (62). Although it has been previously documented in mice that macrophages can convert to an M2 phenotype at the later stages of an M1 response, more recent evidence failed to confirm these findings both in mouse and in human macrophages (63, 64).

As long as the bacterial influx in periodontitis is successfully eradicated, the acute inflammatory process will result either in complete resolution with healing and return of the tissue to its preinflammatory state or elimination of the infection with fibrosis. An alternative outcome includes failure to clear the infection followed by establishment of a chronic inflammatory lesion (65). The above-described pleiotropic functions of macrophages clearly illustrate their central role in regulating the resolution of inflammation and return to homeostasis. The reduction of PMN influx is a critical prerequisite for resolution of inflammation. As previously mentioned, after performing their actions at the inflamed site, PMNs are programmed to undergo apoptosis and are cleared by macrophages (Figure 2), a process called “efferocytosis.” Ingestion of apoptotic PMNs induces changes in the macrophage phenotype and guides the cell toward a resolution phase phenotype (M2 phenotype). This “proresolving” macrophage secretes reparative mediators and anti-inflammatory cytokines, such as IL-10, transforming growth factor-β, and vascular endothelial growth factor, promoting tissue regeneration and return to homeostasis (66). Apoptotic PMNs also secrete mediators, which inhibit leukocyte infiltration. Additionally, since the migration of PMNs would persist in the presence of chemokines, macrophage-specific Metalloproteinase 12 is produced, resulting in the degradation of chemokines with a consequent reduction in leukocyte recruitment (67). As discussed above, incubation with RvE1 reduced L-selectin and CD18 expression in peripheral blood monocytes, indicating that the tissue homing of monocytes is hampered (52). It might thus be plausible to hypothesize that apart from circulating monocytes, M2 macrophages might also originate from tissue-resident or already-recruited macrophages (62, 68).

Isolated macrophages from patients with LAgP exhibit impaired phagocytosis compared to those isolated from healthy controls. After 15 min incubation with 1 nM of RvE1, the phagocytic capacity of LAgP macrophages was enhanced to a level that was similar to that of healthy controls, indicating a possible rescue of the phagocytic defect (50). In addition, when RvE1 was introduced into the peritoneum of rats with zymosan-induced peritonitis, a new subset of proresolving macrophages was identified. These macrophages secreted significantly lower levels of TNF-α, IL-1β, and IL-10 upon stimulation with LPS, compared to other macrophage subpopulations, indicating that RvE1 may reduce the production of proinflammatory cytokines by macrophages *ex vivo* (69).

### Effects on Bone Metabolism

Failure to resolve inflammation within gingival tissue results in the expansion of the inflammatory infiltrate adjacent to alveolar bone. Bone resorption and formation are regulated by the interplay between the receptor activator of nuclear factor-kappa B ligand (RANKL) expressed by OBs, the RANKL receptor (RANK) on OC precursor cells and the soluble decoy receptor osteoprotegerin (OPG) (70). In addition to its expression on OBs, RANKL is also expressed by other cell types, including T- and B-lymphocytes. In the case of unresolved periodontal inflammation, the consequent production of cytokines, chemokines and other mediators stimulates the increased expression of RANKL by these cells. When the expression of RANKL outweighs that of OPG, it allows RANKL to bind RANK on OC precursors and induce the differentiation of pre-OCs to mature OCs, thereby favoring inflammation-induced bone resorption (71). Multiple clinical studies have confirmed that in periodontitis patients, the RANKL/OPG ratio in gingival tissues and gingival crevicular fluid is higher than in healthy controls (72).

Evidence provided by Herrera et al. has shown that nanomolar doses of RvE1 inhibit RANKL-induced OC growth and differentiation, thus downregulating bone resorption *in vitro* (73). This action is mainly mediated through binding of RvE1 to the BLT1 receptor expressed on OCs. Interestingly, in contrast to RvE1, addition of similar doses of EPA to OC cultures increased OC formation, implying that not EPA but one of its metabolic derivatives (RvE1) acts directly on these cells to exert the abovementioned actions. In addition, *via* binding to ChemR23 expressed on OBs, RvE1 increases the generation of OPG by OBs and limits bone resorption *in vitro* (74). The contribution of this lipid mediator to promoting bone formation and inhibiting bone resorption was also confirmed *in vivo* in a rabbit ligature-induced periodontitis model (16). Prevention of *Porphyromonas gingivalis*-induced periodontitis in rats by local application of RvE1 solution was investigated in a 6-week experiment. The results showed that topical treatment with RvE1 prevents and resolves ligature-induced periodontitis with regeneration of soft and hard periodontal tissues to their predisease condition. DNA checkerboard analysis was also performed to investigate changes at the level of periodontal microbiota through the course of the experiment. In the presence of *P. gingivalis*, other bacteria in the biofilm increased as well. After the ligature, *P. gingivalis* application stopped, *P. gingivalis* disappeared from the biofilm, and the resident microflora returned to its normal levels in the RvE1 treated group. However, in the placebo group, the inflammation-induced periodontal breakdown progressed, and the microflora including *P. gingivalis, Actinobacillus actinomycetemcomitans*, and *Fusobacterium nucleatum* became more complex (16, 19). Apart from the possible regenerative potential of RvE1, such a finding also confirms the ecological plaque hypothesis proposed by Marsh; according to this hypothesis, the microenvironment conditions, and more specifically the inflammation level, triggers and determines the composition of the microflora (13). The results of the ligature-induced periodontitis study were recently confirmed in a rat model (17).

### Studies on the Role of Other Proresolving Lipid Mediators

In addition to RvE1, other proresolving mediators, mainly lipoxins, were employed in several studies of periodontal inflammation. Lipoxins are generated by individual LOs (5-LO, 12-LO, and 15-LO) *via* trans-cellular biosynthesis, act within a local microenvironment and are rapidly enzymatically inactivated (75). A significant contribution to the understanding of their proresolving actions originates from several *ex vivo* and *in vivo* animal studies. Lipoxins were shown to counter PMN responses during inflammation, to prevent periodontal bone loss and to promote regeneration in the presence of existing periodontitis (22, 76). Activated PMNs isolated from the peripheral blood of localized juvenile periodontitis patients but not from healthy controls appeared to produce lipoxin A4 (LXA4). The same lipid compound was detected in the gingival crevicular fluid of localized juvenile periodontitis patients, suggesting a possible immunomodulatory role within the local inflammatory milieu of the periodontium (77). Higher levels of lipoxins produced by transgenic rabbits overexpressing 15-LO have been associated with protection against bone resorption when the animals were subjected to ligature-induced periodontitis with *P. gingivalis*. In contrast, non-transgenic rabbits exhibited significant soft- and hard-tissue destruction. Along these lines, when the rabbits were treated with lipoxin stable analogs and subjected to ligature-induced periodontitis, they exhibited markedly reduced bone loss and local inflammation (78). Similarly, ATL lipoxin analogs inhibited recruitment of leukocytes stimulated by *P. gingivalis* in a different *in vivo* model (77).

In addition to their direct proresolving effect of reducing leukocyte infiltration, SPMs have also been associated with indirect roles. Lipoxins promoted the endogenous biosynthesis of other SPMs, such as resolvins, and vice versa, thus amplifying the resolution signaling (76, 79). In *ex vivo* studies of LAgP, the administration of another SPM, Maresin 1, enhanced bacterial killing and restored the phagocytic defect of LAgP phagocytes (80).

The application of benzo-LXA4, combined with surgical treatment of osseous defects in domesticated miniature pigs, resulted in the formation of new alveolar bone, new connective tissue attachment and new cementum, providing a promising field for research on periodontal regeneration (76). Another link between SPMs and tissue regeneration was provided for the first time by research conducted on human periodontal ligament stem cells (hPDLSCs). HPDLSCs have been characterized as important players in tissue regeneration, and recent evidence has confirmed their ability to biosynthesize SPMs, including resolvin D1, D2, D5, and D6, protectin D1, maresins, and LXB4, thereby uncovering new properties of these cells (81). Along this line, LXA4 induced the proliferation, migration, and wound-healing capacity of hPDLSCs acting through ALX/FPR2 (81).

### Studies on the Role of PUFAs Supplementation in Periodontal Inflammation

A large number of studies conducted during the last two decades have focused on the benefits of dietary PUFAs in the treatment of inflammatory conditions. The results from short-term studies providing individuals with ω-3 fatty acid supplements demonstrated reductions in IL-1β and TNF-α synthesis in cell cultures. In one of the studies, the reduction was more dramatic in older compared to younger women (82, 83).

Regarding periodontal inflammation, a pilot study in which PUFA supplementation was provided as a monotherapy in the treatment of experimental gingivitis for 21 days failed to demonstrate a significant benefit of the dietary supplementation (84). Similar findings were reported for a clinical trial in which subjects with chronic periodontitis received oral hygiene instructions in conjunction with 3 g of PUFAs daily (test group) or oral hygiene instructions combined with placebo for 28 weeks (control group). No significant improvements were demonstrated with respect to clinical outcomes when PUFAs were provided as a monotherapy for chronic periodontitis (85). However, when combined with non-surgical treatment of periodontitis [scaling and root planning (SRP), dietary supplementation with ω-3 fatty acids significantly reduced the gingival index, probing depth, and clinical attachment gain compared to SRP combined with placebo (86)].

Based on the potential synergy of action reported for ω-3 PUFAs and aspirin, El-Sharkawy et al. evaluated the combination of low-dose aspirin together with PUFA supplementation and SRP. The test group received dietary supplementation of fish oil (900 mg of EPA + DHA) and 81 mg of aspirin in combination with SRP, whereas the control group was treated with SRP and a placebo. Significant improvements in clinical periodontal parameters, with reductions in the mean probing depth and in the number of residual deep pockets, were demonstrated in the test group (87). In another randomized controlled trial, Naqvi et al. compared the effect of DHA supplementation and low-dose aspirin (test group) with the daily consumption of placebo (control group) in patients with moderate periodontitis who did not receive any SRP for 3 months. The authors concluded that the test group presented with significantly decreased mean pocket depth and gingival index, in comparison to the control group (88). Finally, Elkhouli et al. (89) evaluated the additional benefit of a 6-month daily PUFA supplementation and low-dose aspirin in conjunction with regenerative therapy in class II furcation defects. At 6 months, the test group presented a significantly greater reduction in mean probing pocket depth (0.9 mm) and gain in clinical attachment level (0.6 mm) compared to the control group (89). Apart from the improvement in clinical parameters, the experimental protocol achieved a host modulatory effect reflected by a significant reduction in the levels of proinflammatory markers, such as IL-1b, in the gingival crevicular fluid (88, 89). In the absence of a third group, which should have used ω-3 fatty acids as a monotherapy, the positive results cannot be attributed to PUFA alone, since long-term aspirin use in patients with periodontitis was found to be associated with significantly lower mean values of periodontal attachment loss compared to periodontal patients who did not receive this medication (90).

Although these findings open a promising pharmacologic avenue for an adjunct to periodontal treatment, the limited number of subjects, the variability in the dose of PUFAs within studies, the short-term follow-up and the non-investigated possible side effects are few of the limitations of the abovementioned studies. One possible side effect of ω-3 fatty acids was reported in the late 1970s by Dyerberg and Bang, who demonstrated that the Greenlandic Inuits had significantly prolonged bleeding times compared with healthy Danes. The authors proposed that ω-3 fatty acids affect platelet function and aggregation, a finding later confirmed by other studies (91). However, according to later studies, such prolongation of bleeding time did not exceed normal limits and did not result in clinically significant bleeding in patients using high doses of ω-3 fatty acids alone or in combination with antiplatelet or anticoagulant therapies (92, 93).

## Discussion and Conclusion

Inflammatory response is a protective biological process designed to eliminate the harmful stimulus and promote return of the tissue to homeostasis. Resolution of inflammation is a well-orchestrated active process mediated by a variety of specialized lipid mediators. RvE1 is biosynthesized from EPA and selectively interacts with specific receptors to inhibit further leukocyte infiltration and cytokine/chemokine generation, to induce the apoptosis of PMNs and their removal by macrophages and to restore tissue homeostasis.

In periodontitis, as well as in other inflammatory conditions, inflammation fails to resolve and results in chronic pathology. Recent studies have detected lower levels of SPMs in the stimulated whole blood of LAgP patients, suggesting a defect, which might contribute to the failure of resolution (50). A growing body of *in vitro* and *in vivo* evidence points to the effects of RvE1 and other SPMs on different cell types in regulating the resolution of periodontal inflammation. To date, no study has addressed the issue of the tissue-expression levels of SPMs in gingival biopsies retrieved from chronic gingivitis patients, as opposed to periodontitis patients. With this important piece of information lacking, it is yet unknown whether a robust engagement of the resolution pathways can efficiently prevent the conversion of gingivitis to periodontitis.

To compensate for rapid metabolism or dilution of proresolving lipid mediators, SPM-stable analogs have been constructed as mimetics of endogenous resolution, offering new therapeutic approaches. In addition, incorporation of SPMs in microparticles and the employment of nanoproresolving medicines have provided a new possibility for local delivery at the site of inflammation (76). Although promising results have been obtained in animal studies, the efficacy of this experimental data is yet to be established in human clinical trials.

Human studies involving ω-3 fatty acid supplementation and low-dose aspirin as adjunct to periodontal treatment provide promising results and indicate a synergistic effect of these agents in periodontal treatment. Up to now, there have been no long-term randomized clinical trials investigating the clinical benefits of ω-3 fatty acids versus other broadly used pharmacological agents, such as antibiotics, as an adjunct to periodontal treatment. Furthermore, large scale *in vivo* and *ex vivo* studies evaluating the effects of treatment with RvE1 in individuals with periodontitis may shed more light into the complex molecular mechanisms involved in the resolution of periodontal inflammation. Further studies are warranted to elucidate if RvE1, alone or in combination with other regimens, is a legitimate candidate for periodontitis therapy.

## Author Contributions

MB and EN conceived, wrote the manuscript, and designed the figures. BL participated in the writing and revised the manuscript for final corrections. All authors approved the final version of the manuscript for publication.

## Conflict of Interest Statement

The authors declare that the research was conducted in the absence of any commercial or financial relationships that could be construed as a potential conflict of interest.

## Abbreviations

ALX/FPR2, formyl peptide receptor 2; BLT1, leukotriene B4 receptor; ChemR23, receptor for chemerin and Resolvin E1; COX2, cyclooxygenase 2; DHA, docosahexaenoic acid; EPA, eicosapentaenoic acid; hPDLSCs, human periodontal ligament stem cells; IL, interleukin; LAgP, localized aggressive periodontitis; 5-LO, 5-lipoxygenase; LTB4, leukotriene B4; LXA4, lipoxin A4; LXB4, lipoxin B4; OB, osteoblast; OC, osteoclast; OPG, osteoprotegerin; PAMP, pathogen-associated molecular pattern; PMN, polymorphonuclear neutrophil; PUFAs, polyunsaturated fatty acids; RANK, receptor activator of nuclear factor kappa-B; RANKL, receptor activator of nuclear factor kappa-B ligand; ROS, reactive oxygen species; RvE1, Resolvin E1; SPMs, specialized proresolving mediators; TNF-α, tumor necrosis factor alpha.
